# Body Anthropometry and Bone Strength Conjointly Determine the Risk of Hip Fracture in a Sideways Fall

**DOI:** 10.1007/s10439-020-02682-y

**Published:** 2020-11-12

**Authors:** Marco Palanca, Egon Perilli, Saulo Martelli

**Affiliations:** 1 Department of Industrial Engineering, School of Engineering and Architecture, Alma Mater Studiorum – Università di Bologna, Bologna, Italy; 2 Department of Oncology and Metabolism, and INSIGNEO Institute for in silico Medicine, University of Sheffield, Sheffield, UK; 3 Medical Device Research Institute, College of Science and Engineering, Flinders University, Adelaide, Australia; 4 School of Mechanical, Medical and Process Engineering, Queensland University of Technology, Brisbane, Australia

**Keywords:** Femur, Sideways fall, Fracture mechanics, Strain analysis, High-speed testing

## Abstract

We hypothesize that variations of body anthropometry, conjointly with the bone strength, determine the risk of hip fracture. To test the hypothesis, we compared, in a simulated sideways fall, the hip impact energy to the energy needed to fracture the femur. Ten femurs from elderly donors were tested using a novel drop-tower protocol for replicating the hip fracture dynamics during a fall on the side. The impact energy was varied for each femur according to the donor’s body weight, height and soft-tissue thickness, by adjusting the drop height and mass. The fracture pattern, force, energy, strain in the superior femoral neck, bone morphology and microarchitecture were evaluated. Fracture patterns were consistent with clinically relevant hip fractures, and the superior neck strains and timings were comparable with the literature. The hip impact energy (11 – 95 J) and the fracture energy (11 – 39 J) ranges overlapped and showed comparable variance (CV = 69 and 61%, respectively). The aBMD-based definition of osteoporosis correctly classified 7 (70%) fracture/non-fracture cases. The incorrectly classified cases presented large impact energy variations, morphology variations and large subcortical voids as seen in microcomputed tomography. In conclusion, the risk of osteoporotic hip fracture in a sideways fall depends on both body anthropometry and bone strength.

## Introduction

Osteoporotic fractures are a burden for the public health costing 37 billion Euros in EU and 16 billion Dollars in USA every year.^6^,^24^ Hip fractures occur when the hip load generated at the hip while falling exceeds its load bearing capacity, or strength.^5^ Hip strength is a function of femur morphology, bone mineral density (BMD) and bone architecture^43^,^45^,^47^ whereas the hip load experienced during a fall relates to the dynamics of the fall, the body size and shape, the stiffness of the hip, of its surrounding tissues and of the floor.^4^,^46^,^49^ Yet, the interdependent role of hip strength and fall dynamics in osteoporotic hip fractures is unclear.

According to the World Health Organization (WHO), the diagnosis of osteoporosis and the consequent clinical indication for treatment of patients at risk of fracture is typically based on Dual-Energy X-ray Absorptiometry (DXA) measurements of areal Bone Mineral Density (aBMD), which represents a surrogate of hip strength.^8^,^26^–^28^,^51^ However, the diagnosis of osteoporosis has shown 59% sensitivity and 75% specificity in identifying fracture from non-fracture cases,^8^ motivating research into advanced diagnostic methods. For example, finite-element calculation of hip strength, taking in input the 3D morphology and BMD distribution of the hip from computed tomography (CT) images, showed 6–7% improved accuracy over corresponding aBMD measurements alone.^50^ Other authors developed multivariate statistical models using a variety of parameters, such as BMD, age, history of fragility fracture, premature menopause, oral corticosteroid use and ethnicity, showing 83% sensitivity and 54% specificity.^8^,^26^ Therefore, a large fraction of people at risk of hip fracture are currently not getting preventive fracture treatment, while several other healthy individuals are being treated despite their moderate risk of hip fracture.

Biomechanical studies of hip fracture have most often focused either on hip strength or fall dynamics, separately. Several hip fracture studies used relatively low loading rates (impact speed ranging from 15 to 50 mm/s, with test performed in material testing machines)^13^,^52^ or high-energy dynamic tests (impact speed approximately 114 mm/s, with a drop tower driven by gravity acceleration)^16^,^19^,^23^ showing that hip strength is a function of BMD, morphology and loading rate. Some authors suggested that the highly variable hip bone microstructure observed in high-resolution CT images of osteoporotic femoral heads may play an important role in determining hip strength.^2^ Other studies^4^,^32^ focused specifically on the fall dynamics, showing that the hip load at touch down is a function of body anthropometry (height and weight), stiffness of the hip, the nearby soft-tissues, the pelvis and the floor. Yet, to the best of the authors’ knowledge, only a few studies have replicated hip fractures *in vitro* by accounting for variations of body fall dynamics and compliance of the soft tissues surrounding the hip,^16^–^19^ showing that the compliance of the soft tissues surrounding the hip is an important co-factor for the risk of fracture of the hip.^19^

Here, we hypothesize that variations of body anthropometry, conjointly with the bone strength, determine the risk of hip fracture. As such, we aimed at comparing, in a cohort of donors, the variation in impact energy due to differences in body anthropometry, to the variation of hip fracture energy during a simulated sideways fall. A novel drop-tower experiment was developed for varying the impact energy according to the donor anthropometry. Ten femurs from elderly donors spanning a large range of hip strength, body height, weight and Body Mass Index (BMI) were tested. The impact force, displacement and strain at the superior femoral neck were determined. Variations of impact energy were compared to variations in fracture energy, fracture load and to factors affecting hip strength such as aBMD, bone morphology and bone spatial distribution. Finally, the classification of osteoporosis based on aBMD (T-score) was compared between fracture and non-fracture cases in the experiment. False-positive and false-negative cases were analysed and discussed.

## Material and Methods

### Femur Specimens

Ten fresh-frozen human cadaver femurs, without the soft-tissues, were obtained from elderly Caucasian donors through a body donation program in USA (Science Care, Phoenix, USA), who collected written informed consent. Exclusion criteria included donors younger than 45, bone cancer and interruption of the normal ambulation for longer than one year before death. Both genders were accepted. The range of body height, weight and age at death were, respectively, 142–185 cm, 32–136 kg and 56–91 years (Table 1). Specimens were stored at – 20 °C at the Biomechanics and Implants Laboratory of Flinders University (Tonsley, South Australia, Australia) ensuring the mechanical properties of the specimens were properly maintained.^29^ Ethics clearance was obtained from the institutional Social and Behavioural Research Ethics Committee (SBREC 6380).

**Table 1:** Donor’s anthropometry and femoral dimensions.

ID	Gender	Age	Body weight (kg)	Height (cm)	BMI (kg/m^2^)	Femoral length (mm)	Head radius (mm)	Neck radius (mm)	Neck length (mm)	Anteversion angle (°)	Neck-shaft angle (°)
1	F	56	116	163	44	369	18	20	56	10	137
2	F	81	78	168	28	385	18	18	37	− 40	105
3	M	63	95	185	28	N/A	26	N/A	N/A	N/A	N/A
4	F	70	136	155	57	392	23	16	48	− 11	125
5	F	68	118	163	45	399	22	15	47	− 1	130
6	F	79	58	170	20	437	22	12	60	2	121
7	F	91	57	142	28	415	22	14	45	− 8	122
8	F	77	32	150	14	370	20	11	46	17	122
9	F	78	88	152	38	362	20	13	45	− 3	123
10	F	48	42	157	17	N/A	21	N/A	N/A	N/A	N/A

**Figure 1:**
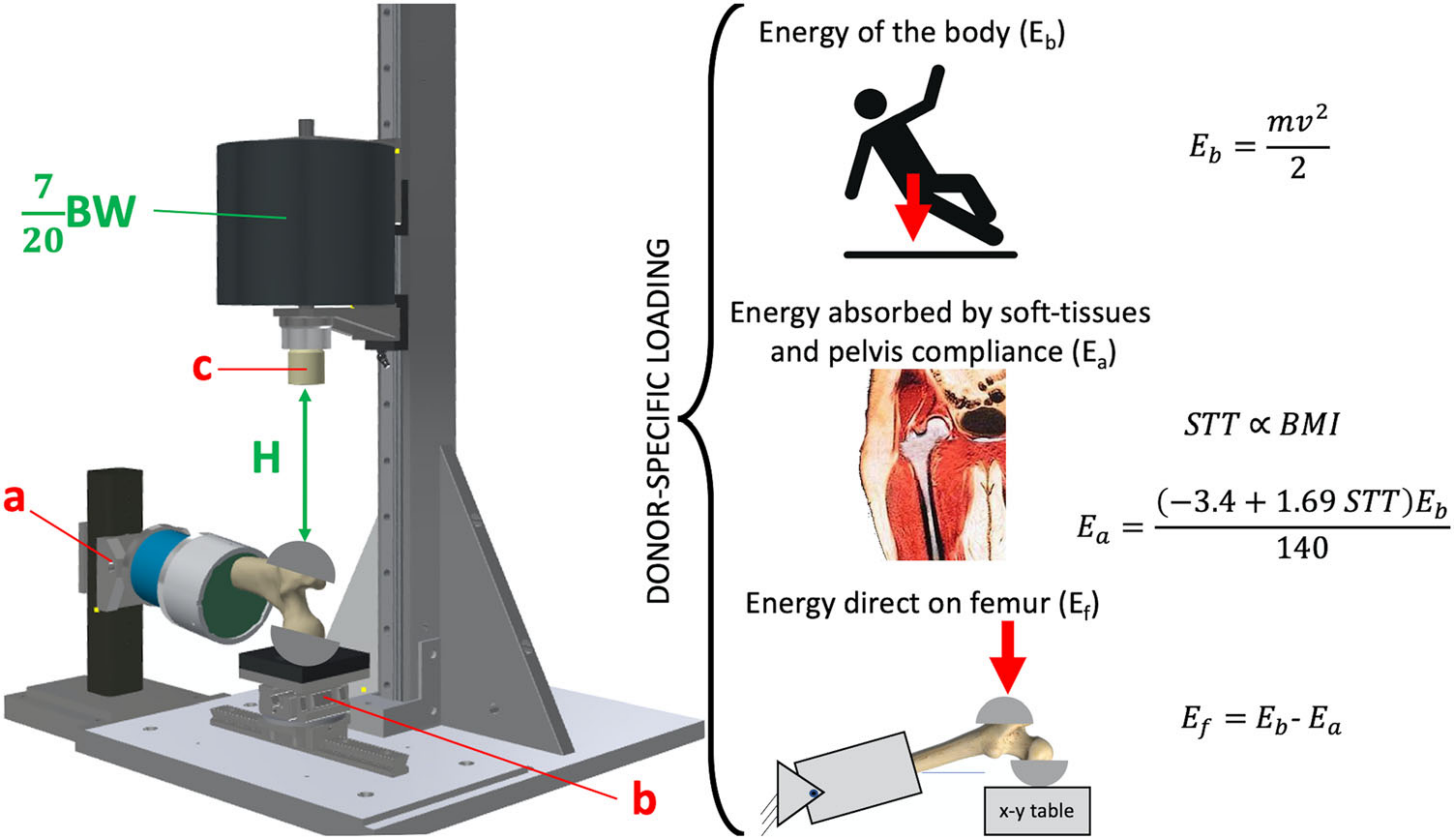
On the left-hand side, the drop tower machine. The aluminium plate and the distal hinge joint (a), the low-friction *x*–*y* table (b), the hammer equipped with the impact load cell (c) are displayed. On the right-hand side, a schematic representation of the process used for determining the drop height and mass including the jack-knife model (top), the energy balance at the hip (centre) and the schematic representation of the simulated impact on the femur (bottom).

The diaphysis of each femur was cut at 180 mm from the top of femoral head and the soft tissues were removed. The diaphysis was abducted by 8° and potted 55 mm deep in a cylindrical aluminium cup using dental cement (Vertex Self Curing, Vertex-Dental, The Netherlands). Two polished aluminium hemi-spherical caps were fixed to the femoral head and the greater trochanter using a 5 mm-thick layer of dental cement to distribute the load on the bone, prevent local crushing and minimize friction (Fig. 1). The specimens and the hammer were painted using a white-on-black speckle pattern.^42^ A white water-based homogeneous paint was sprayed as a background over the entire proximal femur and the hammer. A black water-based paint was used to create random speckles 4–15 pixels in size (1 pixel = ca. 0.05 mm), so that the speckle size was smaller than the facet size for the Digital Image Correlation (DIC) analysis (15–19 pixels).^42^

### Determination of Impact Energy, Drop Mass and Height

The drop tower mass and height were determined using the donors anthropometry and thickness of the soft-tissues pad surrounding the hip.^7^,^16^ The body was simulated as a two-link 45° jack-knife model. The total kinetic energy at touchdown was modelled as a function of the portion (seven-twentieth) of the body weight acting on the hip and the body height according to the equation by van den Kroonenberg et al.^49^:

1 $$E_{\text{b}} = \frac{1}{2} \times \left( {\frac{7}{20}{\text{BW}}} \right) \times \left( {2.72 \times \sqrt h } \right)^{2}$$

where *BW* is the patient’s body weight expressed in kilograms, *h* is the body height expressed in meters and *E*_b_ is the total energy of the impact expressed in Joules. The energy absorbed by the soft-tissue and pelvis (*E*_a_) was determined as a function of the soft-tissue thickness (STT) by using the relationship by Robinovitch et al.^46^:

2 $$E_{\text{a}} = \frac{{\left( { - 3.4 + 1.69 \times {STT}} \right) \times E_{\text{b}} }}{140}$$

The soft-tissue thickness was estimated using the donors’ BMI and a regression equation based on a cohort of men and women (*STT* = 3.4 × BMI − 44.77) within 19–29 BMI range.^7^ Since the fall energy absorbed by the soft-tissue *E*_a_ necessarily tends to *E*_b_ as BMI increases, we assumed a fixed value of the energy absorbed by the soft tissues for BMI greater than 29. The energy taken by the femur (*E*_f_) was then the difference between the total energy generated during the fall (*E*_b_) and the energy absorbed by the soft tissue (*E*_a_). The drop height of the hammer was calculated as follows:

3 $$H = \frac{{M/E_{\text{f}} }}{g}$$

where *H* is the dropping height of the hammer in meters, *g* the gravity acceleration (9.81 m/s^2^) and *M* the drop mass (7/20 BW).

### The Drop-Tower Experiment

The drop-tower machine (Fig. 1) consisted of a base aluminium plate, a vertical beam and a slider mounted on low-friction ball-bearing rails. The hammer hosted a modular mass (5–50 kg) and was equipped with a uniaxial piezoelectric impact load cell (KPA MNC, Kelba, Australia). The load cell was plugged to a data logger (cDAQ-9188, National Instruments, USA). The specimen was fixed to the base of the drop tower machine through a hinge, which allowed the specimen to rotate freely in the quasi-frontal plane. The specimen diaphysis was abducted by 10° and internally rotated by 15° with respect to the loading axis, mimicking the average femoral pose during a fall on a side.^1^ The position of the distal constraint was adjusted to align the longitudinal axis of the impact load cell (hammer) to the greater trochanter. The cap of the femoral head was placed in the centre of a low-friction horizontal *x*–*y* table.

The impact force on the greater trochanter was recorded at 20 kHz and low pass filtered using a zero lag 3rd-order recursive Butterworth filter (3 kHz cut-off frequency).^20^ The hammer position and the superior femoral neck surface were video-recorded at 20,000 fps using two high-speed cameras (Phantom v1212, Vision Research, USA). The drop mass was unfastened manually. The load data and DIC acquisitions were triggered using a photoelectric sensor placed immediately above the impact region. The displacement of the hammer and the strain on the superior surface of the femoral neck were measured from the recorded high-speed cameras images with a three-dimensional DIC algorithm (Istra4D, Dantec Dynamics, Denmark). Before each test, DIC calibration was performed using a calibration target provided by the manufacturer. The DIC procedure was optimized and validated earlier, providing a spatial resolution of 3 mm and a strain random error below 200 microstrain, using a 15–19 pixels facet size, 5 pixels grid spacing, a 5 × 5 contour smoothing and a validity quote of 55%.^40^,^41^

**Figure 2:**
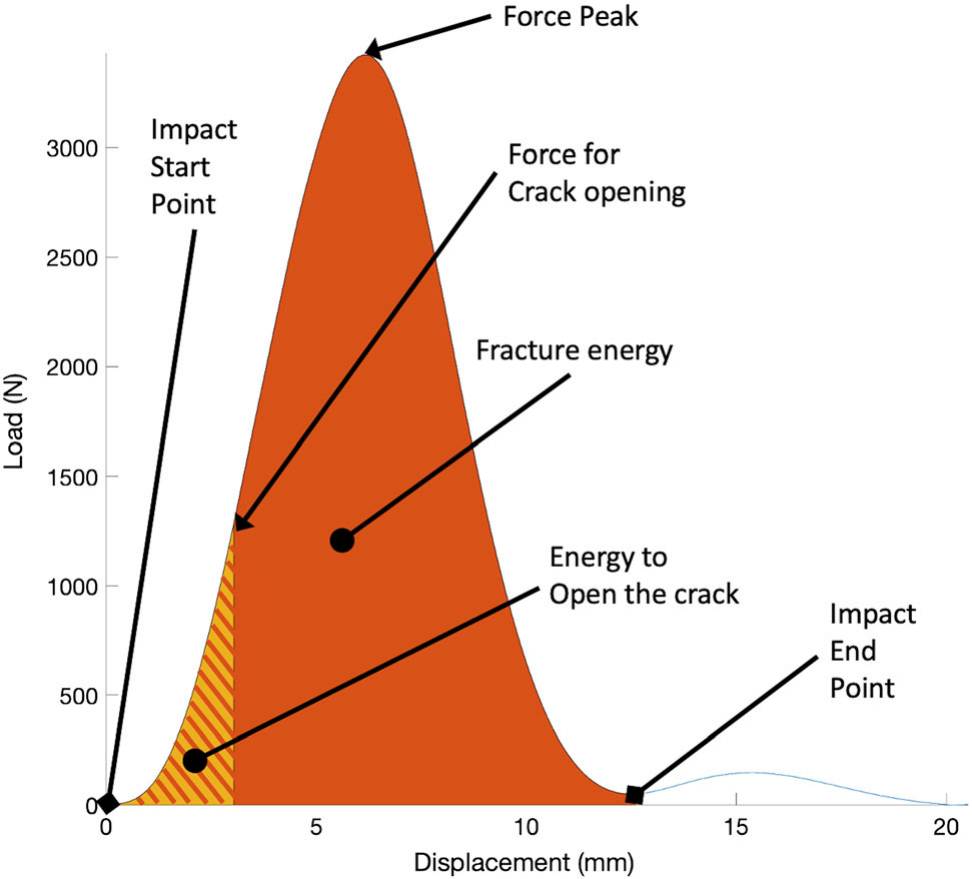
A representative load-displacement curve (specimen #2). The force at crack opening, the peak force, the total fracture energy (sum of the orange and striped portions) and the energy at crack opening (stripped portion) are displayed.

The peak force and the force at crack opening were obtained from the load cell measurements. The impact start time was defined as the point when the impact force raised over 10 N and the impact end time when the force profile reached the first minimum value after decreasing the force below 10% of the max force. The time at crack opening was visually identified from the high-speed video. The position of the hammer was tracked by the DIC, using the hammer position at touchdown (start time) as reference for the displacement measurement. The energy absorbed by the specimen was calculated as the integral of the force over the displacement. The total energy and the energy at the time of crack opening were calculated (Fig. 2).

### Clinical Classification of Fracture Risk

The hip aBMD and the corresponding T-score values were estimated from CT scans. In summary, specimens and a densitometry calibration phantom (QCT Pro, Mindways, USA) were scanned using a CT scanner (Optima CT660, General Electric, USA); the phantom comprised five dipotassium hydrogen phosphate (K_2_HPO_4_) samples (equivalent density range 58.88–157.13 mg cm^−3^). The grey-levels in the CT images were calibrated to equivalent voxel-by-voxel bone density levels in the phantom. The hip bone mass was calculated from the calibrated CT images, then projected on the frontal plane. The aBMD was calculated using the bone mass and the area of the hip projected in the frontal plane^3^ and then converted into equivalent T-score values using the regression equations by Khoo *et al*.^31^ Specimens were categorized into normal (T-score > − 1), osteopenic (− 1 > T-score > − 2.5) and osteoporotic (T-score < − 2.5).

### Microstructural Imaging

The microarchitecture of the specimens was imaged using a high-resolution synchrotron-light micro-CT (SR-microCT, Australian Synchrotron, Clayton VIC, Australia) using an earlier *ad-hoc* imaging protocol.^36^ Briefly, the X-ray projection images were acquired using a dedicated detector (2560 × 2160 pixels) and off-axis scanning mode, resulting in scanned and reconstructed field of view equal to 145.7 × 145.7 × 131.4 mm at an isotropic voxel size of 0.03 mm.

### Analysis of the Data

The ability of the new protocol to reproduce clinically relevant hip fractures was assessed, by comparing (1) the fracture pattern to the AO/OTA fracture classification^38^ and (2) the fracture mechanics (time to fracture, force at crack opening, peak force, principal tensile and compressive strain maps) to corresponding published values.^12^,^17^,^23^,^52^ We also evaluated tensile, compressive and shear strain maps at the location of the fracture onset in the high-speed video, to ascertain whether shear maps are a better indicator of fracture onset location than principal tensile and compressive strain as observed in an earlier study on fracture of metastatic vertebrae.^39^

As we hypothesize that variations of body anthropometry, conjointly with the bone strength, determine the risk of hip fracture, we expect the variation of the energy delivered to the femur while falling and the variation of the hip fracture energy to overlap. Over the ten specimens, the range and coefficients of variation (standard deviation/average) of the total impact energy due to changes in body anthropometry and of the hip fracture energy were compared.

The normality of the distributions was verified using a Shapiro-Wilk test. Pearson’s correlation was used to identify relationships between T-score, total impact energy, energy delivered to the femur, hip fracture energy and fracture load (Prism 8, GraphPad Software, USA). Statistical significance was set to 0.05.

Specimens at high and low risk of facture were categorized using the threshold T-score equal to − 2.5, below which patients are considered at high risk of hip fracture according to the current WHO guidelines. False-positive and false-negative cases were analysed by comparing the impact energy and T-score. The effect of hip morphology on fracture was assessed by comparing the lever arm between the two points of contact on the femoral head and the greater trochanter, using quasi-frontal projection images of the micro-CT scans. The presence of large bone microstructural defects, such as large bone voids,^22^ at the fracture onset location, was ascertained in the microstructural images by visualizing 5 mm thick quasi-frontal micro-CT slices through the fracture onset location using Drishti software.^33^

## Results

**Figure 3:**
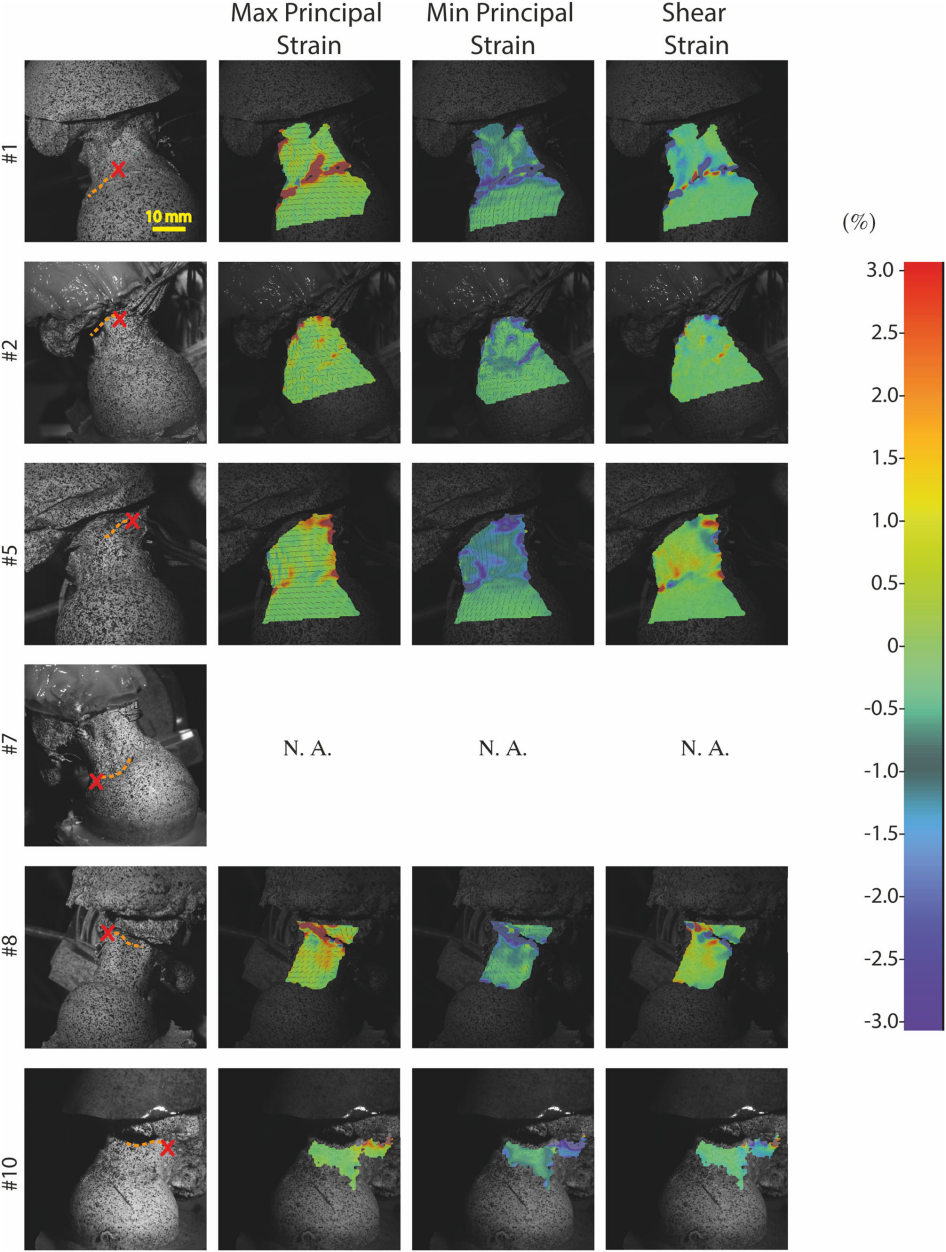
High-speed camera images with strain maps obtained by applying DIC. The fracture onset location (red cross, first column) and the crack pattern (orange dotted lines), the principal tensile (second column), the principal compressive (third column) and the shear strain prior to crack opening (fourth column). The images of specimen #6 and the strain maps for specimen #6 and #7 are not available.

Seven femurs out of ten fractured and displayed pertrochanteric and subcapital fracture patterns, according to the AO/OTA fracture classification.^38^ The peak force was 2984 ± 913 N (average ± standard deviation) (Table 2). The time to fracture onset and to peak force were 2.7 ± 0.9 ms and 4.1 ± 5.1 ms, respectively. Fractured specimens showed local tensile strains on the neck surface at the fracture onset location and compressive strains over a large portion of the superior neck exceeding 3% (Fig. 3). Of the specimens that fractured, the strain map at the location of fracture onset was available for three specimens (#1, #5, #8), and the maximum shear strain gradient was visually closer and more localized around the fracture onset location than the principal strains. For the non-fractured specimens, strain reached 0.3 – 0.5% both in tension and compression.

**Table 2:** Comparison between the classifications of fracture cases based on aBMD (T-score) and the observed fractures types.

ID	AO/OTA fracture	T-score	Fracture classification	M (kg)	H (cm)	*STT* (mm)	F_c_ (N)	F_p_ (N)	T_c_ (ms)	T_p_ (ms)	*E*_b_ (J)	*E*_a_ (J)	*E*_f_ (J)	E_c_ (J)	E_fr_ (J)
1	31B1.1	0.77	0 (FN)	41	4	80	2844	3489	2.9	3.8	244	230	14	3.2	11
2	31A1.2	− 1.03	0 (FN)	27	27	49	1281	3423	1.9	3.1	168	96	72	1.3	18.2
3	NF	− 1.06	1	33	29	50	–	2912	–	4.1	228	133	95	–	–
4	NF	− 1.62	1	48	3	80	–	3315	–	13.4	273	257	16	–	–
5	31B2.3	− 2.74	1	41	4	80	1772	2289	2.7	4.2	249	235	14	1.8	8.6
6	^b^	− 3	1	20	48	23	1212	1230	3.3	15.7	128	33	95	^a^	^a^
7	31B1.2	− 3.09	1	20	22	51	1988	2999	2.3	3.3	104	61	43	2.8	14.5
8	31B2.2	− 3.28	1	11	56	3	110	1320	0.6	2.4	62	1	61	0.06	18.4
9	NF	− 3.59	0 (FP)	31	3	80	–	2969	–	4.4	174	163	11	–	–
10	31A2.2	− 3.9	1	15	51	13	3530	3976	2.7	13.0	85	11	74	9.3	39

The correct classification of fracture cases is indicated by 1, the incorrect classification is indicated by 0 and is separated in false-negative (FN) and false-positive (FP) cases

Italicized physical quantities identify estimated results

*E*_b_, total energy; *E*_a_, energy absorbed by the soft tissues; *E*_f_, E_c_ and E_fr_, nominal energy taken by the femur, to open the crack and to fracture the femur, respectively; M, drop mass; H, height of the hammer; *STT*, soft tissue thickness; F_c_, force at the first crack opening; F_p_, peak force; T_c_, time at crack opening; T_p_, time to peak force; NF, non fracture cases

^a^Missing data

^b^Not possible to reconstruct the fracture type because of the several fragments after fracture

The soft tissue absorbed the majority (122 ± 96 J) of the total impact energy (172 ± 75 J) reducing the energy delivered to the femur to 50 ± 34 J. The energy taken by the femur had a coefficient of variation of 69% (34 J/50 J), which is comparable to the coefficient of variation of the T-score in the same cohort (CV = 65%). For the seven fractured specimens, the energy taken by the femur at crack opening was 3.1 ± 3.2 J (CV = 103%) and the energy to fracture the femur was 18 ± 11 J (CV = 61%). For the three specimens that did not fracture, the energy taken at the time of peak force was 14 − 30 J. The T-score was significantly and positively correlated to the total energy (*r* = 0.65, *p*-value = 0.04). A positive trend was observed between the T-score and the energy absorbed by the soft tissues (*r* = 0.55, *p*-value = 0.10) while a negative trend between the T-score and the fracture energy (*r* = − 0.51, *p*-value = 0.30). No correlation was found between the T-score and the energy delivered to the femur (*r* = − 0.13, *p*-value = 0.72).

**Figure 4:**
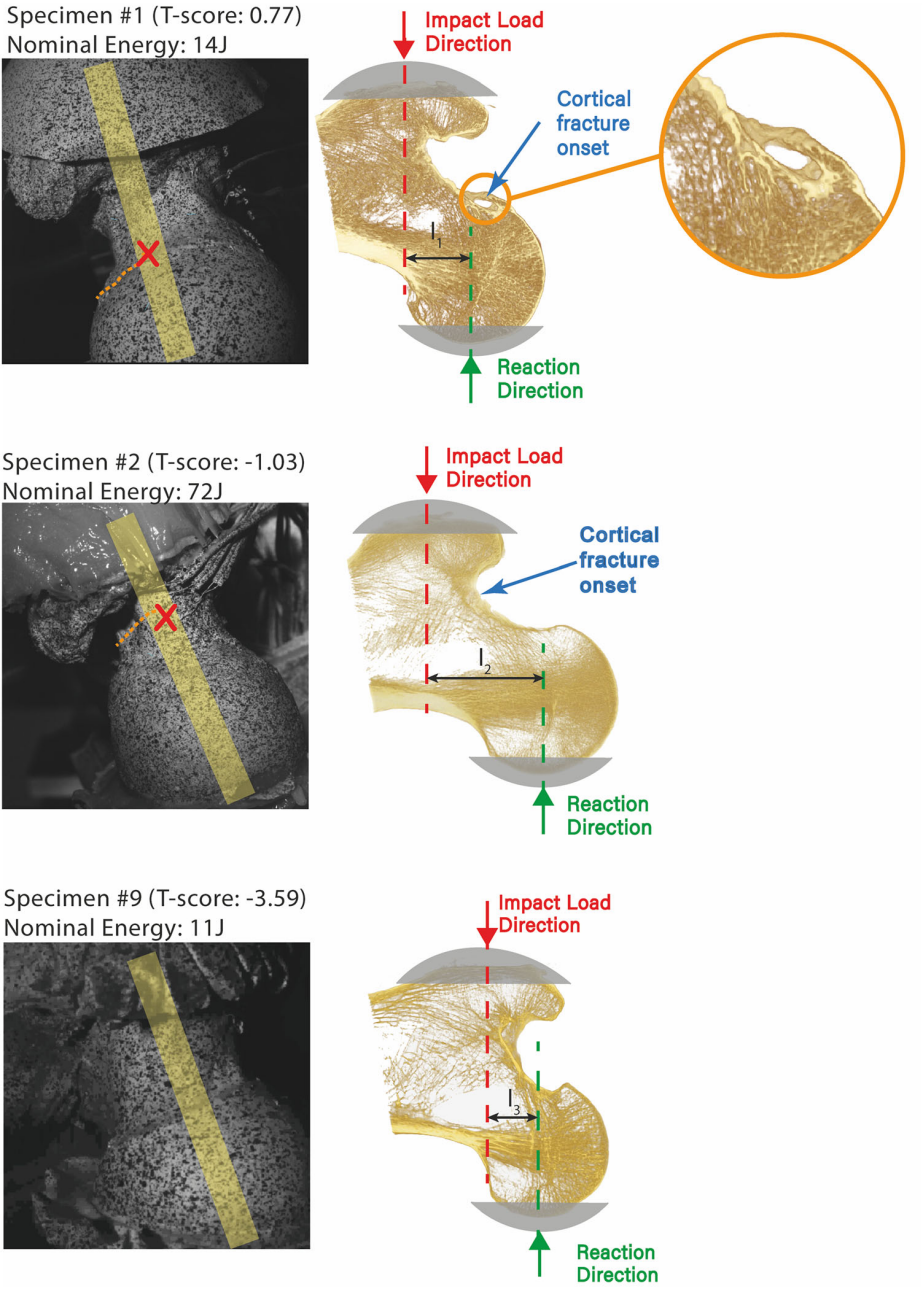
The femur microarchitecture visualized by micro-CT (0.03 mm voxel size) shown as a 5 mm thick quasi-on a quasi-frontal cross section for the false-negative (#1 and #2) and false-positive (#9) cases. The location of the slice is displayed (yellow shaded region) superimposed to the high-speed images for DIC taken during the drop-tower experiment, together with the cortical fracture onset (red cross) and pattern (orange dotted lines). The loading condition of the impact is also schematically displayed in the microarchitectural images. The l indicates the lever arm between the impact load direction and the reaction direction, #2 had a larger lever arm compared to #1 and #9.

The aBMD in the specimens spanned the lower range for the Caucasian population (T-score = -2.3 ± 1.5). Specimens included six osteoporotic, three osteopenic and one normal femur. The presence of osteoporosis correctly classified fracture from non-fracture for 7 of the 10 cases. One normal specimen (#1, T-score = 0.77) did fracture (false-negative) under a 14 J impact energy (*E*_b_ = 244 J; *E*_a_ = 230 J; height = 163 cm; weight = 116 kg; BMI = 44). One osteopenic specimen (#2, T-score = − 1.03) fractured (false-negative) using a nominal impact energy equal to 72 J (*E*_b_ = 168 J; *E*_a_ = 96 J; height = 168 cm; weight = 78 kg; BMI = 28). One osteoporotic specimen (#9, T-score = − 3.59) did not fracture (false-positive) under 11 J impact energy (*E*_b_ = 174 J; *E*_a_ = 163 J; height = 152 cm; weight = 88 kg; BMI = 38). There was a variable distance between the two contact points on the femoral head with the *x*–*y* table and the greater trochanter with the load cell across the false-negative and false-positive cases (Fig. 4). Specimen #1, which had a normal aBMD (T-score = 0.77), displayed a large subcortical void at fracture onset location. No evident signs of major subcortical voids were observed in specimens #2 and #9.

## Discussion

The aim of this study was to develop a drop-tower testing protocol for replicating the dynamics of hip fracture in elderly adults to test the hypothesis that variations in body anthropometry and bone strength determine the risk of hip fracture. The variation of the hip impact energy caused by variation of body anthropometry was compared to that of hip fracture energy. The experimental fracture and non-fracture cases were analysed with respect to the classification of fracture and non-fracture cases using the T-score threshold (*T* < − 2.5, osteoporosis). We found that (a) the novel drop-tower protocol produces clinically relevant hip fractures; (b) the variation across individuals of impact energy at the hip while falling on a side overlaps with that of the hip fracture energy; and (c) variation in impact energy, bone morphology and the presence of major subcortical bone voids can limit the ability of the classification of fracture and non-fracture cases using only the T-score definition of osteoporosis. As such, body anthropometry contributes to determine the risk of hip fracture in osteoporosis. Nevertheless, bone morphology and major subcortical bone voids determine fracture load (strength) and energy, and may also co-contribute to the low sensitivity and specificity of the current clinical practice for diagnosing patients at high risk of hip fracture.

The hip fracture patterns were consistent with the AO/ATA hip fracture classification,^38^ and the superficial neck strains and timing in the experiment were consistent with earlier reports,^17^,^23^ confirming a realistic fracture dynamic in the experiment. Interestingly, the shear strain map, when available, better indicated the location of fracture onset than principal strain maps, possibly indicating a different failure mechanism^48^ than the opening in tension or crushing in compression most often used for fracture prediction.^12^,^37^ The fracture load (range 1230–3976 N) was in the lower range of that reported in the literature (1170–7601 N),^12^,^17^,^23^,^52^ likely because the specimens in the present study were in the lower range of aBMD distribution in the elderly population (aBMD: 0.4–1.0 mg/cm^2^) compared to those reported in the aforementioned studies (aBMD: 0.4–1.85 mg/cm^2^).^12^,^17^,^23^,^52^ Therefore, the present results apply to the part of the population most at risk of hip fracture.

The range of energy delivered to the femur (11–95 J) overlapped with that of the hip fracture energy (8.6–39 J). Variation of body anthropometry (body weight, height and BMI) caused a coefficient of variation of impact energy and load over the sample (CV = 61–103%) comparable to that of aBMD (CV = 61%). Therefore, variation of body inertia, determined by body anthropometry, is an important co-factor in determining the risk of hip fracture in elderly individuals. This is in agreement with earlier findings obtained with a different setup and protocol,^17^ hence showing the limit of fracture prediction based on aBMD alone. We also found that the majority of the fall energy can be absorbed by the soft-tissues and that the total fall energy and the energy absorbed by the soft-tissues are correlated with the aBMD, as an epidemiological study has shown.^34^ This result is in agreement with the known negative correlation between BMI and risk of fracture,^14^,^21^ the notion that the soft-tissue thickness plays a fundamental role in establishing the risk of fracture^4^,^7^,^45^ absorbing the largest part of the energy in the hip region^16^ and the effectiveness of hip protectors in preventing femur fractures.^9^,^25^ However, the energy delivered to the femur was not correlated to the T-score. As such, T-score and body anthropometry are independent factors that, conjointly, determine the risk of hip fracture.

There was a surprising agreement between the ability of the T-score to predict fracture in the present experiment (70% accuracy) and that reported in the clinical literature (59–75%).^8^,^11^,^26^–^28^,^51^ Among the three cases not correctly classified using the definition of osteoporosis, we observed a highly variable impact energy. Specimen (#1), showing normal aBMD, fractured under a low-energy impact (14 J). An osteopenic specimen (#2), also not considered at high risk of hip fracture according to the definition of osteoporosis, fractured but after being subjected to a high-energy impact (72 J); that specimen showed no major subcortical voids. The osteoporotic specimen (#9), which was expected to fracture under a low-energy impact (11 J), did not fracture. We also observed a major subcortical void (#1) and a variable moment arm (#1, #2, #9) between the impact force on the trochanter and the reaction force on the femoral head. Thus, these findings support the notion that microstructural weak points^22^,^43^ and hip morphology can influence the fracture risk.^15^,^35^ Therefore, it appears that multi-factorial models accounting for body anthropometry, bone mass, bone spatial distribution and morphology may help improving current classification techniques in osteoporosis.

One limitation of the present study is the relatively low number of specimens (*n* = 10), which may limit the generality of our findings. For example, the small size prevents from analysing differences in hip fracture dynamics due to donor gender, age, bone morphology and microstructure. More research is necessary to address this point. It is important to note that in the present study, the soft-tissue thicknesses were estimated using a regression equation, possibly raising concerns about how well the soft-tissue thickness was represented for each donor. However, studying the individual risk of fracture in each single donor was not an aim of this study. The present study provides the first experimental evidence of the effect of body anthropometry on hip fracture mechanics in a cohort of elderly people spanning the lower range of bone density and exhibiting a large variation in body anthropometry. We also show that the fracture and non-fracture cases that were misclassified by the definition of osteoporosis do show specific energy, bone morphological and microstructural features, hence, supporting earlier (and future) studies investigating these aspects. Another limitation resides in the single representative loading configuration.^30^ The effect of body anthropometry on the risk of hip fracture reported here may vary for different falling scenarios. Finally, the propensity to fall, which is a function of neuromotor health condition^44^ and the related potential protective effect of muscle contraction,^10^,^16^ determines the likelihood that a fall event or fracture will occur. The present study analysed the effect of hip strength, in relation to its morphology, bone density and the presence of large microstructural defects, such as large bone voids (as seen in microcomputed tomography), and fall dynamics (as a function of body anthropometry) independently from the propensity to fall. Further retrospective and prospective studies are necessary to demonstrate the validity of the conjoint use of body anthropometry and hip strength for fragility prediction in clinics.

In conclusion, body anthropometry is an independent co-factor in hip fracture dynamics and may help improving fracture prediction in osteoporosis.

## References

[1] Askarinejad S, Johnson JE, Rahbar N, Troy KL (2019). Effects of loading rate on the of mechanical behavior of the femur in falling condition. J. Mech. Behav. Biomed. Mater..

[2] Augat P, Schorlemmer S (2006). The role of cortical bone and its microstructure in bone strength. Age Ageing.

[3] Bauer JS, Henning TD, Müeller D, Lu Y, Majumdar S, Link TM (2007). Volumetric quantitative CT of the spine and hip derived from contrast-enhanced MDCT: conversion factors. Am. J. Roentgenol..

[4] Bhan S, Levine IC, Laing AC (2014). Energy absorption during impact on the proximal femur is affected by body mass index and flooring surface. J. Biomech..

[5] Bhattacharya P, Altai Z, Qasim M, Viceconti M (2019). A multiscale model to predict current absolute risk of femoral fracture in a postmenopausal population. Biomech. Model. Mechanobiol..

[6] Blume SW, Curtis JR (2011). Medical costs of osteoporosis in the elderly Medicare population. Osteoporos. Int. J. Establ. Result Coop. Eur. Found. Osteoporos. Natl. Osteoporos. Found. USA.

[7] Bouxsein ML, Szulc P, Munoz F, Thrall E, Sornay-Rendu E, Delmas PD (2007). Contribution of trochanteric soft tissues to fall force estimates, the factor of risk, and prediction of hip fracture risk. J. Bone Miner. Res..

[8] Chen SJ, Chen YJ, Cheng CH, Hwang HF, Chen CY, Lin MR (2016). Comparisons of different screening tools for identifying fracture/osteoporosis risk among community-dwelling older people. Med. U. S..

[9] Cheung AM, Detsky AS (2008). Osteoporosis and fractures: missing the bridge?. JAMA.

[10] Choi WJ, Cripton PA, Robinovitch SN (2015). Effects of hip abductor muscle forces and knee boundary conditions on femoral neck stresses during simulated falls. Osteoporos. Int..

[11] Cosman F, de Beur SJ, LeBoff MS, Lewiecki EM, Tanner B, Randall S, Lindsay R (2014). Clinician’s guide to prevention and treatment of osteoporosis. Osteoporos. Int..

[12] Dall’Ara E, Luisier B, Schmidt R, Kainberger F, Zysset P, Pahr D (2013). A nonlinear QCT-based finite element model validation study for the human femur tested in two configurations in vitro. Bone.

[13] de Bakker PM, Manske SL, Ebacher V, Oxland TR, Cripton PA, Guy P (2009). During sideways falls proximal femur fractures initiate in the superolateral cortex: evidence from high-speed video of simulated fractures. J. Biomech..

[14] De Laet C, Kanis JA, Odén A, Johanson H, Johnell O, Delmas P, Eisman JA, Kroger H, Fujiwara S, Garnero P, McCloskey EV, Mellstrom D, Melton LJ, Meunier PJ, Pols HAP, Reeve J, Silman A, Tenenhouse A (2005). Body mass index as a predictor of fracture risk: a meta-analysis. Osteoporos. Int..

[15] Ferris BD, Kennedy C, Bhamra M, Muirhead-Allwood W (1989). Morphology of the femur in proximal femoral fractures. J. Bone Jt. Surg. Br..

[16] Fleps I, Enns-Bray WS, Guy P, Ferguson SJ, Cripton PA, Helgason B (2018). On the internal reaction forces, energy absorption, and fracture in the hip during simulated sideways fall impact. PLoS ONE.

[17] Fleps I, Fung A, Guy P, Ferguson SJ, Helgason B, Cripton PA (2019). Subject-specific ex vivo simulations for hip fracture risk assessment in sideways falls. Bone.

[18] Fleps I, Guy P, Ferguson SJ, Cripton PA, Helgason B (2019). Explicit finite element models accurately predict subject-specific and velocity-dependent kinetics of sideways fall impact. J. Bone Miner. Res..

[19] Fleps I, Vuille M, Melnyk A, Ferguson SJ, Guy P, Helgason B, Cripton PA (2018). A novel sideways fall simulator to study hip fractures ex vivo. PLoS ONE.

[20] George E (2012). Control System Design Guide.

[21] Gonnelli S (2014). Obesity and fracture risk. Clin. Cases Miner. Bone Metab..

[22] Grassi L, Kok J, Gustafsson A, Zheng Y, Väänänen SP, Jurvelin JS, Isaksson H (2020). Elucidating failure mechanisms in human femurs during a fall to the side using bilateral digital image correlation. J. Biomech..

[23] Helgason B, Gilchrist S, Ariza O, Chak JD, Zheng G, Widmer RP (2014). Development of a balanced experimental—computational approach to understanding the mechanics of proximal femur fractures. Med. Eng. Phys..

[24] Hernlund E, Svedbom A, Ivergård M, Compston J, Cooper C, Stenmark J, McCloskey EV, Jönsson B, Kanis JA (2013). Osteoporosis in the European Union: Medical management, epidemiology and economic burden: a report prepared in collaboration with the International Osteoporosis Foundation (IOF) and the European Federation of Pharmaceutical Industry Associations (EFPIA). Arch. Osteoporos..

[25] Järvinen TLN, Sievänen H, Khan KM, Heinonen A, Kannus P (2008). Shifting the focus in fracture prevention from osteoporosis to falls. BMJ.

[26] Kanis JA (2002). Diagnosis of osteoporosis and assessment of fracture risk. Lancet.

[27] Kanis JA, Black D, Cooper C, Dargent P, Dawson-Hughes B, De Laet C, Delmas P, Eisman J, Johnell O, Jonsson B, Melton L, Oden A, Papapoulos S, Pols H, Rizzoli R, Silman A, Tenenhouse A, on behalf of the International Osteoporosis Foundation and the National Osteoporosis Foundation (2002). A new approach to the development of assessment guidelines for osteoporosis. Osteoporos. Int..

[28] Kanis JA, Johnell O, Oden A, De Laet C, Jonsson B, Dawson A (2002). Ten-year risk of osteoporotic fracture and the effect of risk factors on screening strategies. Bone.

[29] Kaye B (2012). The effects of freezing on the mechanical properties of bone. Open Bone J..

[30] Keyak JH, Skinner HB, Fleming JA (2001). Effect of force direction on femoral fracture load for two types of loading conditions. J. Orthop. Res..

[31] Khoo BCC, Brown K, Cann C, Zhu K, Henzell S, Low V, Gustafsson S, Price RI, Prince RL (2009). Comparison of QCT-derived and DXA-derived areal bone mineral density and T scores. Osteoporos. Int..

[32] Laing AC, Robinovitch SN (2010). Characterizing the effective stiffness of the pelvis during sideways falls on the hip. J. Biomech..

[33] Limaye, A. Drishti: a volume exploration and presentation tool. *Proceedings SPIE*, 2012. 10.1117/12.935640.

[34] Lloyd JT, Alley DE, Hawkes WG, Hochberg MC, Waldstein SR, Orwig DL (2014). Body mass index is positively associated with bone mineral density in US older adults. Arch. Osteoporos..

[35] Maeda Y, Sugano N, Saito M, Yonenobu K (2011). Comparison of femoral morphology and bone mineral density between femoral neck fractures and trochanteric fractures. Clin. Orthop. Relat. Res..

[36] Martelli S, Perilli E (2018). Time-elapsed synchrotron-light microstructural imaging of femoral neck fracture. J. Mech. Behav. Biomed. Mater..

[37] Martelli S, Pivonka P, Ebeling PR (2014). Femoral shaft strains during daily activities: implications for atypical femoral fractures. Clin. Biomech..

[38] Müller ME, Nazarian S, Koch P, Schatzker J (1990). The Comprehensive Classification of Fractures of Long Bones.

[39] Palanca M, Barbanti-Bròdano G, Cristofolini L (2018). The size of simulated lytic metastases affects the strain distribution on the anterior surface of the vertebra. J. Biomech. Eng..

[40] Palanca M, Brugo TM, Cristofolini L (2015). Use of digital image correlation to investigate the biomechanics of the vertebra. J. Mech. Med. Biol..

[41] Palanca M, Marco M, Ruspi ML, Cristofolini L (2018). Full-field strain distribution in multi-vertebra spine segments: an in vitro application of digital image correlation. Med. Eng. Phys..

[42] Palanca M, Tozzi G, Cristofolini L (2016). The use of digital image correlation in the biomechanical area: a review. Int. Biomech..

[43] Perilli E, Baleani M, Öhman C, Fognani R, Baruffaldi F, Viceconti M (2008). Dependence of mechanical compressive strength on local variations in microarchitecture in cancellous bone of proximal human femur. J. Biomech..

[44] Phelan EA, Mahoney JE, Voit JC, Stevens JA (2015). Assessment and management of fall risk in primary care settings. Med. Clin. North Am..

[45] Roberts BJ, Thrall E, Muller JA, Bouxsein ML (2010). Comparison of hip fracture risk prediction by femoral aBMD to experimentally measured factor of risk. Bone.

[46] Robinovitch SN, Hayes WC, McMahon TA (1991). Prediction of femoral impact forces in falls on the hip. J. Biomech. Eng..

[47] Seeman E, Delmas PD (2006). Bone quality—the material and structural basis of bone strength and fragility. N. Engl. J. Med..

[48] Tang T, Ebacher V, Cripton P, Guy P, McKay H, Wang R (2015). Shear deformation and fracture of human cortical bone. Bone.

[49] van den Kroonenberg AJ, Hayes WC, McMahon TA (1995). Dynamic models for sideways falls from standing height. J. Biomech. Eng..

[50] Viceconti M, Qasim M, Bhattacharya P, Li X (2018). Are CT-based finite element model predictions of femoral bone strengthening clinically useful?. Curr. Osteoporos. Rep..

[51] WHO (2007). Assessment of Osteoporosis at the Primary Health Care Level.

[52] Zani L, Erani P, Grassi L, Taddei F, Cristofolini L (2015). Strain distribution in the proximal Human femur during in vitro simulated sideways fall. J. Biomech..

